# Use of an anti‐CD200‐blocking antibody improves immune responses to AML *in vitro* and *in vivo*

**DOI:** 10.1111/bjh.17125

**Published:** 2020-09-30

**Authors:** Namrata Rastogi, Sarah Baker, Stephen Man, Robert A. Uger, Mark Wong, Steven J. Coles, Marie Hodges, Amanda F. Gilkes, Steven Knapper, Richard L. Darley, Alex Tonks

**Affiliations:** ^1^ Department of Haematology Division of Cancer & Genetics School of Medicine Cardiff University Cardiff CF14 4XN UK; ^2^ School of Biosciences European Cancer Stem Cell Research Institute Cardiff University Cardiff CF24 4HQ UK; ^3^ Trillium Therapeutics Inc Mississauga ON Canada; ^4^ School of Science and the Environment University of Worcester Worcestershire WR2 6AJ UK; ^5^ Cardiff Experimental and Cancer Medicine Centre (ECMC) School of Medicine Cardiff University Cardiff CF14 4XN UK

**Keywords:** AML, CD200, immune check point, immunosuppression, smouldering multiple myeloma

## Abstract

Treatment of relapsed/resistant acute myeloid leukaemia (AML) remains a significant area of unmet patient need, the outlook for most patients remaining extremely poor. A promising approach is to augment the anti‐tumour immune response in these patients; most cancers do not activate immune effector cells because they express immunosuppressive ligands. We have previously shown that CD200 (an immunosuppressive ligand) is overexpressed in AML and confers an inferior overall survival compared to CD200low/neg patients. Here we show that a fully human anti‐CD200 antibody (TTI‐CD200) can block the interaction of CD200 with its receptor and restore AML immune responses *in vitro* and *in vivo*.

Acute myeloid leukaemia (AML) is a highly heterogeneous disease in which leukaemic stem cell (LSC) persistence is considered to be the primary cause of relapse.^1^ Immune evasion by LSC is an important determinant of relapse which is mediated by expression of specific cell surface molecules with immune modulatory function.^2^ Novel immune‐directed therapeutic approaches form a major focus of current and clinical research.^3^ We have previously shown that CD200 is an important immune checkpoint protein that is expressed in c. 40% of AML patients and associates with poor prognosis.^4^ CD200 belongs to the immunoglobulin superfamily and exerts immunosuppressive signalling through its receptor CD200R, present on immune cells.^5^ We have previously shown that CD200^High^ AML patients exhibited reduced Natural Killer (NK) and T cell immune responses in comparison to CD200^Low^ patients, indicating that CD200 is a potential immunotherapeutic target in this disease. Our results also suggested that CD200 can be a contributing factor responsible for AML immune evasion and therapy relapse.^6^, ^7^, ^8^, ^9^ With this in mind, we used an anti‐CD200‐blocking antibody (TTI‐CD200) to assess whether CD200‐mediated immunosuppression can be reversed in AML. TTI‐CD200 is a fully human antibody that neutralises human CD200 with nanomolar potency, as determined through a cell‐based assay (Figure S1).

Initially, we determined the effective blocking concentration of TTI‐CD200, using K562 cells over‐expressing CD200 (K562‐CD200^+^), by co‐culturing them with normal human NK cells (see Online Supplementary Materials and Methods). As expected, diminished expression of the NK degranulation marker CD107a was observed for K562‐CD200^+^ compared to K562‐CD200^‐^ cells (Figure S2). Co‐culturing K562‐CD200^+^ in the presence of TTI‐CD200 significantly recovered activity of CD3^‐^CD56^dim^CD16^+^ NK cells (equivalent to K562‐CD200^‐^ cells), compared to isotype control (Figure S2A). Similarly, we also observed an increase in Interferon gamma (IFN)‐γ release from NK cells as measured by ELISPOT assay in the presence of TTI‐CD200, compared to isotype‐treated cells (Figure S2B). To test whether TTI‐CD200 could have a functional effect on immune cells *ex vivo*, AML patient blasts with high or low levels of CD200 protein expression (as described in ^6^) were co‐cultured with their autologous lymphocytes in the presence of TTI‐CD200 or isotype control. A significant increase in CD107a expression was observed in TTI‐CD200 treated CD200^High^ AML blasts in comparison with isotype control‐treated cells (Fig. 1A). As expected, no increase in CD107a expression was observed in CD200^Low^ AML blasts treated with TTI‐CD200 or isotype control. TTI‐CD200 treatment also led to significant increase in IFN‐γ release in CD200^High^ AML blasts in ELISPOT assay (Fig. 1B). Our previous studies showed that CD200^High^ AML patients had a low frequency of IFN‐γ producing CD4^+^ Th1 cells (CD19^‐^CD3^+^CD4^+^CCR7^‐^), which are central to adaptive immune responses in AML.^7^ Therefore, we next determined the impact of TTI‐CD200‐blocking on the frequency of these cells in CD200^High^ AML patients. As expected, an increase in frequency of IFN‐γ‐producing CD4^+^ Th1 cells was observed in the presence of TTI‐CD200 (Fig. 1C). Taken together, these data show that an *ex vivo* blockade of the CD200‐CD200R interaction with TTI‐CD200 led to the recovery of a significant proportion of immune activity.

**Fig 1:**
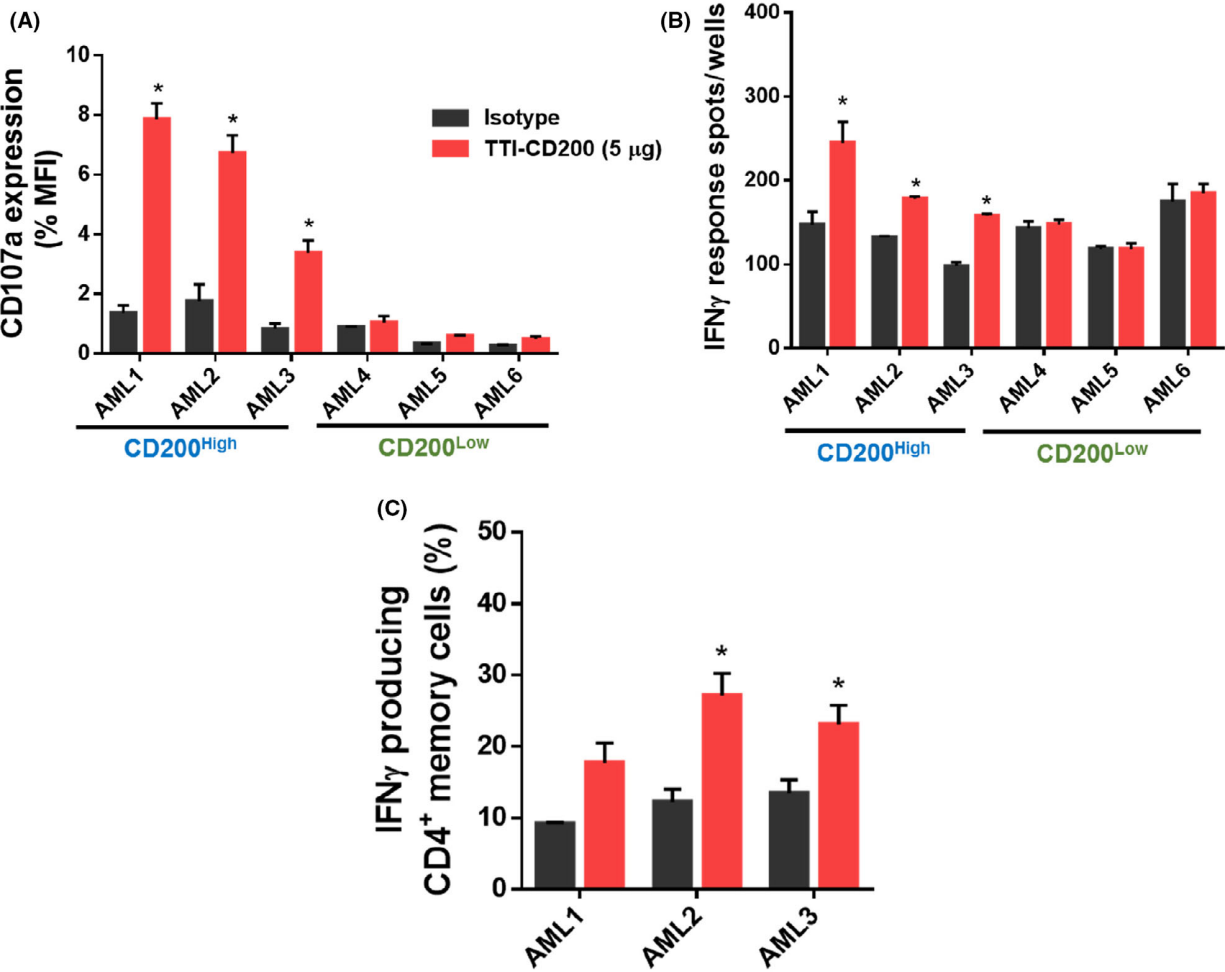
TTI‐CD200 treatment improves immune responses of CD200^High^ AML patient samples: (A) Bar graphs showing NK killer cell activity measured as CD107 expression. Primary AML blasts from CD200^High^ and CD200^Low^ AML samples (previously determined by upper or lower quartile of CD200 expression) with their autologous lymphocytes were treated with either TTI‐CD200 or isotype control (5 μg/mL) for 6h in the presence of Golgi stop. NK cell activity was measured as the surface expression of CD107a and represented as percentage of MFI relative to isotype control. (B) Bar graphs representing spots/wells as a measure of Interferon‐γ (IFN‐γ) production. Primary AML blasts from CD200^High^ and CD200^Low^ AML samples (as above) were incubated with their autologous lymphocytes on ELISPOT plates, pre‐coated with IFN‐γ capture antibody. IFN‐γ capture spots were counted manually under a light microscope and normalised with negative control wells without capture of IFN‐γ antibody. (C) Bar graph showing frequency of active IFN‐ γ‐producing CD3^+^CD4^+^CCR7^‐^ effector memory T cells in CD200^High^ AML samples. AML samples with their autologous lymphocytes were treated with either TTI‐CD200 or isotype control (5 μ g/mL) for 6h, fixed and permeabilised, and stained for intracellular cytokines. The percentage of of IFN‐γ‐producing cells was measured by flow cytometry based on cell surface markers (details in supplementary methods). All datasets are represented as mean ± 1SD from three experiments. Statistical significance is denoted by *P ≤ 0·05, analysed by unpaired ‘*t’* test.

To analyse the effect of TTI‐CD200 *in vivo,* a robust source of adoptive immune cells which could be co‐engrafted with AML blast cells into NOD‐SCID IL2Rγ^(‐/‐)^ (NSG) mice was required. Cytokine Induced Killer (CIK) cells are an adoptive immune cellular therapy which had shown promising pre‐clinical and clinical efficacy in AML, without causing graft‐versus‐host‐like disease which is normally observed with adoptive transferred human T cells in immuno‐deficient mice.^10^ Also, CIK cells are readily expandable and effective at killing tumour cells in a non‐MHC‐restricted manner. We therefore selected CIK cells as our adoptive immune cells to analyse the efficacy of TTI‐CD200 in a xenograft model of AML. Poh and Linn (2016) have also shown that surface expression of immune checkpoint proteins (PD1, TIM3, LAG3, CTLA‐4 and CD200R) decreases their cytotoxicity towards myeloid target cells, which can be reversed using monoclonal antibodies (though CD200 was not tested). Initially, we determined the expression of CD200R on two major cytotoxic populations of CIK cells (CD56^+^CD3^+^ and CD56^‐^CD3^+^). CD200R expression was detectable in these populations at day 10 of culture (Figure S3). To confirm that CIK activity was inhibited by CD200, we co‐cultured CIK cells with K562‐CD200^+^ cells and found that K562‐CD200^+^ cells were less susceptible to CIK‐mediated killing than K562‐CD200^‐^ cells (Figure S4A). Further, pre‐treatment of K562‐CD200^+^ cells with TTI‐CD200 increased CIK cell‐mediated cell lysis compared to isotype control (Figure S4B and S4C) with the cytotoxicity of CIK cells maintained across different effector to target (E:T) ratios (Figure S4D). Interestingly, TTI‐CD200 treatment increased surface expression of CD107a on CIK cells, suggesting activation of the NK cell‐like phenotype.^11^ Together these data show that CD200 negatively regulates the cytotoxic activity of CIK cells. We next confirmed these findings using primary AML blasts *ex vivo*. TTI‐CD200 pretreatment increased CIK cell‐mediated lysis of CD200^High^ AML blasts (Fig 2A). While the therapeutic benefit of CIK cells has already been verified in AML patients, CIK cells expressing chimeric antigen receptors (CAR) may allow enhanced targeting of AML cells,^11^ although identification of AML‐specific antigens will be required. These findings show that inhibition of CD200‐CD200R signalling significantly augments the cytotoxic potential of CIK cells towards AML blasts; CD200 can therefore be considered a good candidate for future CAR‐CIK‐based therapy in AML.

**Fig 2:**
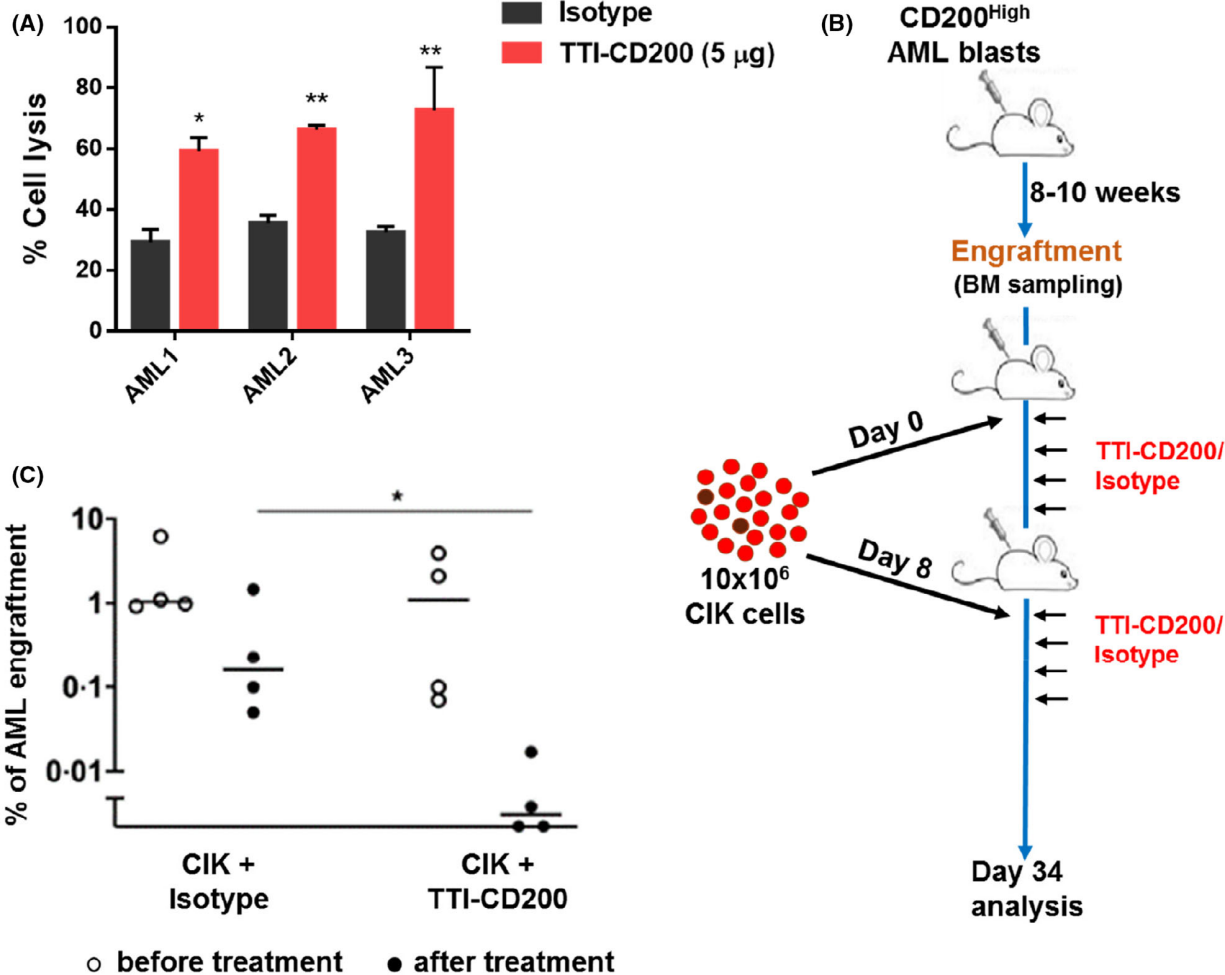
**TTI‐CD200 sensitises AML cells towards CIK cell‐mediated lysis *ex vivo* and *in vivo*.** (A) Bar graphs represent percentage of cell lysis of primary AML blast cells. Cells were pre‐treated with either TTI‐CD200 or isotype control (5 μ g/mL) for 1h, followed by co‐culture with CIK cells for 5h with E:T of 10:1. Other E:T ratios are shown in supplemental Fig. S5. Percentage of cell lysis was calculated using flow cytometry and shows the relative number of AML blasts recovered, following CIK cell co‐culture in TT‐CD200 and isotype‐treated groups. Data are mean ± 1SD from three experiments with * *P* ≤ 0·05 and ** *P* ≤ 0·01, analysed by Dunnett’s multiple comparison test. (B) Schematic plan for *in vivo* experiments. NSG mice were injected with primary CD200^High^ AML blasts. Mice were monitored for 8‐10 weeks for AML engraftment by bone marrow sampling and subsequently injected with CIK cells every seventh day, followed by treatment with TTI‐CD200 or isotype (10mg/kg) on the day, and every two days for eight times. (C) Data represent percentage of AML engraftment in NSG mice as analysed through flow cytometry as the expression of hCD45^+^CD33^+^CD19^‐^. Every point represents an individual mouse. Horisontal bar represents the mean with *P ≤ 0·05, analysed by the Mann‐Whitney test.

The above data suggest that TTI‐CD200 can increase immune activity of CIK cells *ex vivo* by relieving the CD200R immunosuppression signal imparted by AML blasts. As a proof of principle, to determine whether TTI‐CD200 can achieve similar results *in vivo*, we injected human CD200^High^ AML blast cells into NSG mice. Once AML engraftment was achieved, CIK cells were subsequently injected weekly, followed by administration of TTI‐CD200 or Isotype control every two days (Fig 2B). AML engraftment was assessed after eight cycles of antibody treatment. We found a significant decrease in percent engraftment of AML cells in the TTI‐CD200 treatment group compared to isotype control (Fig 2C). Taken together, our findings illustrate for the first time the use of CD200 monoclonal antibody in the context of a xenotransplantation model of AML.

The efficacy of immune checkpoint inhibitors has been well‐established in recent years as they have gained therapeutic approvals for solid tumours and in refractory non‐Hodgkin Lymphoma.^12^ Monoclonal antibodies against immune checkpoint proteins such as CTLA‐4 (ipilimumab), PD‐1 (nivolumab and pembrolizumab) and its ligand PD‐L1 (durvalumab), TIM‐3 (MBG453) are under active clinical investigation in high risk as well as refractory/relapsed AML.^3^ However, a limited number of patients so far have benefited from these agents as monotherapy.^13^ Various further studies on combinatorial approaches have shown moderate but promising clinical benefits to the patients but immune‐related adverse events (irAE) remain a potential concern for this group of therapies.^14^ Therefore, identification of novel immune checkpoint proteins used alone or in combination, which have improved tolerability and clinical efficacy in the refractory/relapse disease setting, is a major focus of current AML research. Though the importance of CD200 in AML prognosis and modulating immune response has already been shown, little evidence of efficacy of an anti‐CD200 monoclonal antibody have been documented in pre‐clinical models of AML to facilitate its clinical development. We have shown that TTI‐CD200 treatment not only enhanced the function of autologous immune cells *ex vivo* but also significantly improved efficacy of adoptive immune effector CIK cells towards residual AML cells *in vivo*. Additional studies suggest that CD200 may be a potential marker for LSCs responsible for relapse in AML and that its targeting can benefit patients with relapsed or refractory AML disease.^15^ Furthermore, significant correlation between CD200 and PDL1 in AML T cell immunosuppression has already been shown by our group, suggesting that this combination also holds potential for further evaluation in AML immunotherapy.^9^ Therefore, we propose that blocking the CD200‐CD200R axis represents a potentially effective strategy to treat AML and warrants further clinical investigation.

## Conflict of Interest

RAU and MW are employees of Trillium Therapeutics Inc.

## Author contributions

NR designed and performed *in vitro* and *ex vivo* experiments, analysed all data and co‐wrote the manuscript. The anti‐CD200‐blocking antibody used in this study was provided by Trillium Therapeutics Inc (RAU and MW). SB, MH, AFG assisted with patient AML blast isolation. SM and SJC provided resources and edited the paper. SK, RLD and AT secured funding and contributed to experimental design, data analysis and co‐wrote the manuscript.

## Supporting information

Figure S1. TI‐CD200 antibody inhibits CD200‐mediated suppression with nanomolar potency. The NK‐sensitive target cell C1R expressing cell surface CD200 was incubated with titrated TTI‐CD200 antibody or isotype control for one hour and then co‐cultured with a human NK.
Figure S2. TTI‐CD200 improves immune responses in CD200^High^ AML cells *in vitro*.
Figure S3. Expression of CD200 receptor on CIK cells.
Figure S4. TTI‐CD200 treatment enhances CIK‐mediated lysis in AML cells *in vitro*.
Figure S5. CD200‐blocking increases CIK‐mediated cell lysis in AML blasts.
