# Supplementary material for: Use of an anti‐CD200‐blocking antibody improves immune responses to AML in vitro and in vivo
Source: Br J Haematol. 2020 Sep 30;193(1):155–9. doi: 10.1111/bjh.17125 (PMC9851282; doi:10.1111/bjh.17125)
Supplement: Supplementary file 1 — Figure S1. TI‐CD200 antibody inhibits CD200‐mediated suppression with nanomolar potency. The NK‐sensitive target cell C1R expressing cell surface CD200 was incubated with titrated TTI‐CD200 antibody or isotype control for one hour and then co‐cultured with a human NK. Figure S2. TTI‐CD200 improves immune responses in CD200High AML cells in vitro. Figure S3. Expression of CD200 receptor on CIK cells. Figure S4. TTI‐CD200 treatment enhances CIK‐mediated lysis in AML cells in vitro. Figure S5. CD200‐blocking increases CIK‐mediated cell lysis in AML blasts. [file BJH-193-155-s001.pdf]

Supplementary material and methods:

Primary cell material and cell culture

Diagnostic bone marrow or peripheral blood samples from AML patients were collected before treatment and following informed consent from patients in accordance with the 1964 Declaration of Helsinki. Patient clinical characteristics are outlined below. Human neonatal cord blood was obtained from healthy full-term pregnancies at the University Hospital of Wales, Cardiff, UK with informed consent and with approval from the South East Wales Research Ethics Committee in accordance with the 1964 Declaration of Helsinki. AML blasts or Peripheral Blood Mononuclear (PBMCs) cells from healthy donors were separated using Ficoll-Hypaque (Sigma-Aldrich, Dorset, U.K.) density gradient centrifugation as previously described.[1] AML blast viability and percentage were determined through flow cytometry using 7-actinomycin D (7AAD) and CD45-FITC (Biolegend, San Diego, CA).

K562 cells overexpressing CD200 were generated through retroviral transduction as described previously.[2,3] K562 cells expressing GFP alone were used as a control. Transduced K562 cells were maintained in RPMI-1640 (Sigma-Aldrich), 10% (*v/v*) FCS (Biosera, East Sussex, U.K.), 2 mM L-glutamine (Sigma-Aldrich) according to recommended conditions at 37°C, 5% CO_2 for all experiments. The genetic identity of the cell lines was confirmed by short tandem repeat (STR) according to the manufacturer/supplier at purchase. Monthly monitoring for Mycoplasma contamination was performed and confirmed using the MycoAlert Detection Kit (Sigma-Aldrich).

Patient Characteristics

Sample number	Sample Type	Age (y)	Sex	Cytogenetics	Mutations tested (NPM1/FLT3-TKD/FLT3-ITD)	Diagnosis	WBC ($x10^9/L$)
AML #1	AML	17	M	46,XY[20]	Wt	De Novo	294
AML #2	AML	65	M	47,XY,+8[5]/46,XY[17]	Wt	De Novo	9
AML #3	AML	54	F	46,XX,?dup(12)(p11p12),idic(14)(p11)[18]/47,idem,+idic(14)[2]	FLT3-ITD	De Novo	148.2
AML #4	AML	56	M	46XY[20]	FLT3-ITD	De Novo	64
AML #5	AML	67	M	46,XX[20]	NPM-Mut	De Novo	42
AML #6	AML	63	F	Failed	Wt	De Novo	46
AML #7	AML	51	M	50,XY,+6,+8,+13,inv(16)(p13q22),+22[10]	FLT3-ITD	De Novo	91.8

Assessment of Natural Killer cell activity

Natural Killer (NK) cell sub-population analysis and activity assay was performed as previously described.[3] Briefly, NK cells were characterized based on cell surface CD16 and CD56 protein expression. For NK cell activity assay, K562-CD200$^+$ cells (or equivalent controls K562-CD200$^-$) were co-cultured with normal PBMCs with effector (E) to target (T) ratio (E:T) of 10:1 and incubated with Golgi Stop and CD107a-FITC (or IgG1κ-FITC) antibody (Biolegend) for 1h at 37°C. Following incubation cells were treated with either TTI-CD200 (1, 5, or 10μg) or Isotype control (10μg) (Trillium Therapeutic Inc.) for a further 6h. Cell surface protein expression (CD3, CD16, CD56) and CD107a was analysed by flow cytometry (see data acquisition and analysis). In order to determine the effect of AML patient blasts on NK cell degranulation (in presence of TTI-CD200/Isotype), cryopreserved AML blasts (of known CD200 expression status) were thawed, washed in pre-warmed RPMI-1640 containing 10% (*v/v*) FCS and 2mM L-glutamine and co-cultured with their autologous lymphocytes and assayed for CD107a expression as above.

Inflammatory Interferon-γ (IFN-γ) response was measured through ELISPOT or ELISA assay as previously described.[3] For ELISPOT assay, AML cells were incubated on ELISPOT plates pre-treated with IFN-γ capture antibody and blocked with a pool of common

antigens (PPP). Cells were incubated for 48h undisturbed in the incubator at 37°C. Interferon- γ spots were developed and counted under a stereo light microscope (x8 magnification) and normalized using negative (with no capture antibody) and positive (activated with Ionomycin) controls.

Analysis of CD4^{+} memory T-cells

Memory CD4^{+} memory T- helper 1 (Th1) cells were analysed through measurement of intracellular cytokines, Interleukin-2 (IL-2), Tumour necrosis factor-α (TNF-α) and INF-γ as described previously.[4] Cytokine response was assessed from PBMNCs or BMMNCs following stimulation with 50 ng/ml phorbol 12-myristate 13-acetate (PMA) and 1 µmol/L Ionomycin, for 5 h at 37ºC/5% CO_2 in the presence of Golgi-stop (1:1500, BD Biosciences) and 10 µg/ml Brefeldin-A (Sigma). Unstimulated cells were used as controls. After incubation cells were stained with AquaLive dead cell exclusion dye (Invitrogen, Paisley, U.K.) and CD3-APC-H7, CD4-Pacific blue, CD45RA-APC and CCR7-PerCP-Cy5.5 (all from Biolegend, Cambridge UK). Cells were then fixed and permeabilized using Fix and Perm solution (BD Biosciences), incubated with TNFα-FITC, IL-2-PE or IFNγ-PC7 (Biolegend) and analysed by flow cytometry (see data acquisition and analysis).

Cytokine Induced Killer (CIK) cell culture

Freshly isolated mononuclear cells from cord blood were cultured in RPMI advanced (Thermo Fisher) supplemented with 10% *v/v* FBS, 2mM L-glutamine and penicillin and streptomycin. Cells were activated with 1000 U/mL of IFN-γ (Peprotech, London, U.K) for 24 h at a density of 3x10^{6} cells/mL at 37°C. The following day cells were treated with 50 ng/mL of OKT3 (Biolegend) and 300 U/mL of Interleukin (IL)-2 (Peprotech) for 3 days at 37°C. On day 4 of culture, cells were sub-cultured to 0.5x10^{6} cells/mL with the addition of fresh 300 U/mL IL-2 every two days. CIK cells were assayed for CD200R expression by flow cytometry

after day 10 of culture. CIK cells from day 10 until day 24 were used for *in vitro* and *ex vivo* short-term killing assay as well as *in vivo* experiments as described below.

CIK cell induced short term-killing assay

AML cells were pre-treated with TTI-CD200 or Isotype control antibodies and co-cultured with CIK cells at different E:T ratios (5:1 and 10:1) for 5-6h. Thereafter cells were collected and stained with anti-CD33 (Biolegend) and 7-AAD (Sigma) to determine absolute counts. Percent cell lysis was calculated from the basal level of CIK cell killing without TTI-CD200 pre-treatment and compared with control isotype antibody treated cells.

Immunodeficient mice studies using CIK cells

All animal experiments were performed under the U.K. Home Office project licence (30/3380). NOD-SCID IL2Rγnull (NSG) mice (The Jackson laboratory, Bar Harbor, ME, USA aged 8 to 12 weeks were irradiated at 3.75 Gys (^{137}Cs source) up to 24h before tail vein injection. $2.5X10^6$ primary $CD200^{High}$ AML cells were intravenously injected per mouse (n=8). Mice were observed for AML engraftment for 8-10 weeks through bone marrow sampling using flow cytometry as previously described.[5] Mice successfully engrafted with AML were intravenously injected with CIK cells ($10x10^6$ /injection/mice) starting at day 12 in culture, every seven days followed by intraperitoneal injection of TTI-CD200 or Isotype control antibodies at 10 mg/Kg in the same day, then every other day. A total of two doses of CIK cells and eight doses of TTI-CD200 or isotype antibody injections were given to mice. All mice were sacrificed after 10 days from the last TTI-CD200 injection and analysed for AML engraftment.

Data acquisition and analysis

Flow data were acquired using an Accuri C6 cytometer (Accuri, St Ives, U.K.), FACS Canto II cytometer or LSRFortessa (BD Biosciences). NK cells were gated using CD3-PerCP5.5 surface marker where $CD3^{-}$ lymphocytes were characterized using CD56-APC and CD16-PE (BD Biosciences), $CD56^{dim}$ $CD16^{+}$ were primarily analysed for degranulation. Relative CD200-PE, CD200R-PE and CD107a-FITC (BD Biosciences) expression was determined by calculating mean fluorescence intensity (MFI); background fluorescence was corrected using respective isotype-matched controls. For memory T-cell analysis, live $CD45RA^{++}CD3^{+}$ T cells were gated on $CD4^{+}$ $CCR7^{-}$ for effector memory T-cell. Intracellular cytokines TNFα-FITC, IL-2-PE or IFNγ-PC7 were measured as MFI and corrected for background using respective isotype. CIK cells were characterised by using CD3-PerCP5.5 and CD56-APC (BD Biosciences) surface markers. For animal studies, mice were sacrificed 10 days after the last treatment and the cells from the bone marrow were harvested. Red blood cells were lysed and an aliquot of cells were immunophenotyped to determine the level and nature of human engraftment by flow-cytometry. Dead cells and debris were excluded using 4,6 diamidino-2-phenylindole (DAPI at 1:2000 from a 200 mg/ml stock) staining and >200,000 DAPI- events were collected using an LSRFortessa (BD Biosciences). Human AML engraftment was quantified as the proportion of live cells that were $CD45^{+}CD33^{+}CD19^{-}CD3^{-}$ (CD45-APC/CD33PE/CD19-FITC/CD3-PerCP5.5; all from BD Biosciences). Flow cytometric data were analysed using FCS express v6 (DeNovo Software, Canada). Data analysis was performed using Graph Pad Prism v6 (GraphPad Software, San Diego, CA, USA). The significance of differences was calculated using ANOVA with Tukey's or Dunnett's test for multiple comparisons and student 't' test for correlation. P-values ≤0.05 were considered significant. For the *in vivo* data the nonparametric Mann-Whitney test was used to calculate statistical significance.

Supplementary Figures:

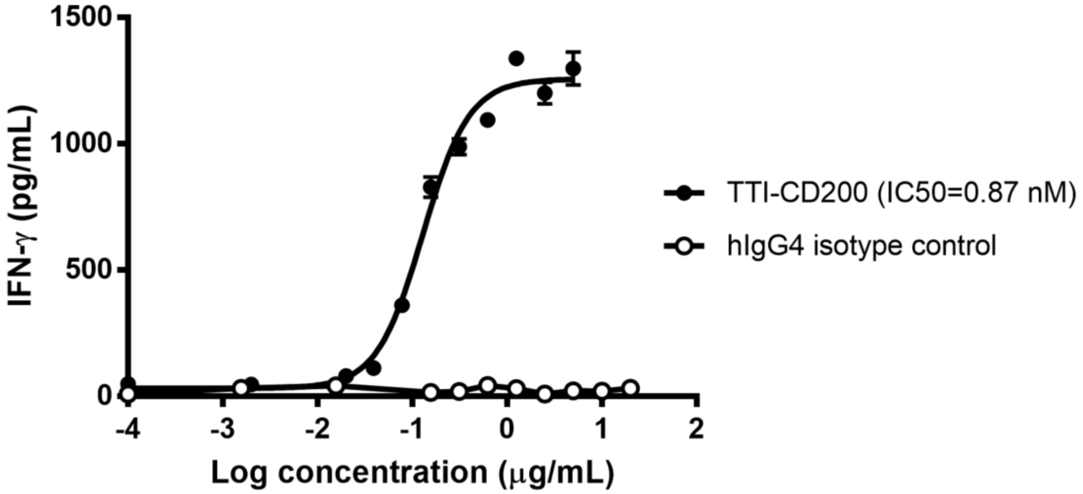

Supplemental Fig. S1. TTI-CD200 Antibody Inhibits CD200-mediated Suppression with Nanomolar Potency. The NK-sensitive target cell C1R expressing cell surface CD200 was incubated with titrated TTI-CD200 antibody or isotype control for 1 hour and then co-cultured with a human NK cell line transfected with hCD200R (NKL-CD200R) for 64 hours. IFN-γ release was measured by ELISA, and the IC50 calculated using a sigmoidal dose-response curve. CD200 on the C1R cells suppresses NKL-CD200R activation (IFN-γ release), which is restored by the anti-CD200 antibody.

(A)

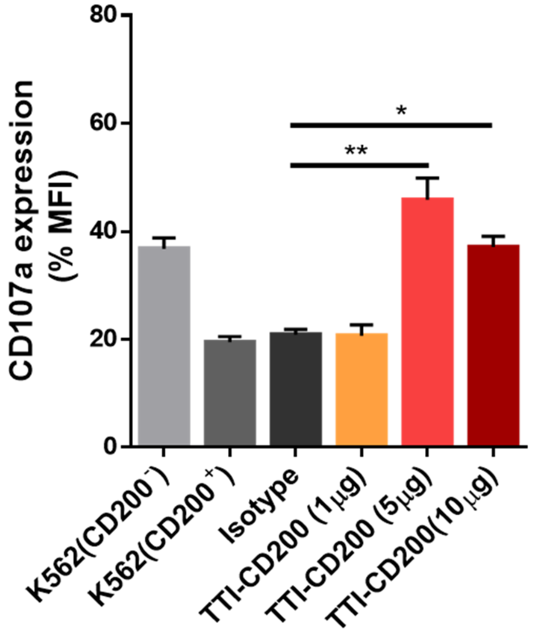

(B)

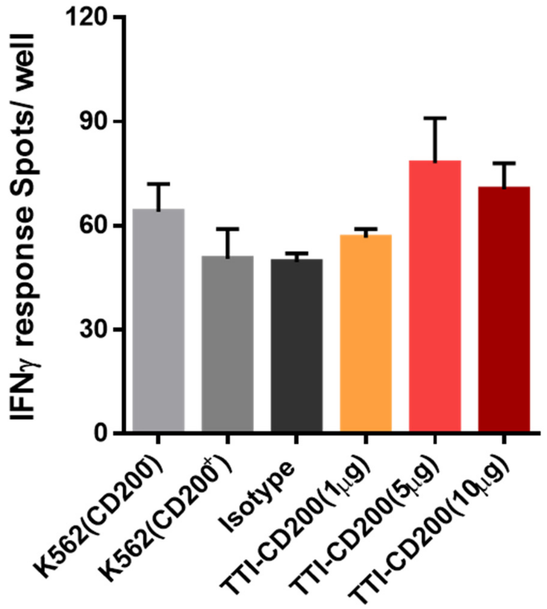

Supplemental Fig. S2: TTI-CD200 improves immune responses in CD200^{High} AML cells *in vitro*. (A) Bar graphs indicating CD107a expression (as percent MFI) as a measure of NK cell activity. K562 cells (CD200$^-$ and CD200$^+$) were co-cultured with normal PBMC in the presence TTI-CD200 at indicated concentrations or Isotype (10μg). Spontaneous activity of normal NK cells without co-culture was used for normalization of CD107a MFI. (B) IFN-γ production measured as spots/well through ELISPOT assays. K562 cells were co-cultured with normal PBMCs in the presence of TTI-CD200 at different indicated concentrations or Isotype (10μg) on ELISPOT plates for 48h. Spots were developed and counted and compared with isotype. Data are mean ± 1SD from three independent experiments. Statistical significance denoted by *$p<0.05$, **$p<0.01$ analysed by Tukey's multiple comparison test.

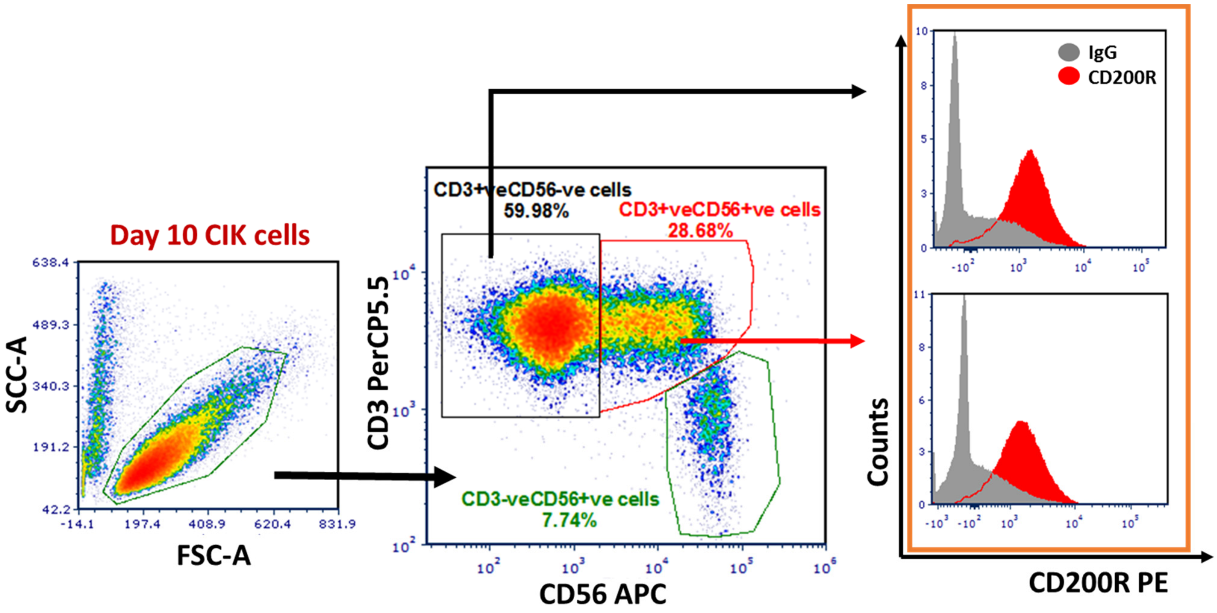

Supplemental Fig. S3. Expression of CD200 receptor on CIK cells. CIK cells were cultured for 10 days and cellular heterogeneity was analysed through flow cytometry based on different cell surface markers for NK and T cell phenotypes. Expression of CD200R was determined on two major and cytotoxic subpopulations of CIK cells, $CD3^{+}$ $CD56^{-}$ (60%) and $CD3^{+}CD56^{+}$ (89%) indicated in black and red regions respectively.

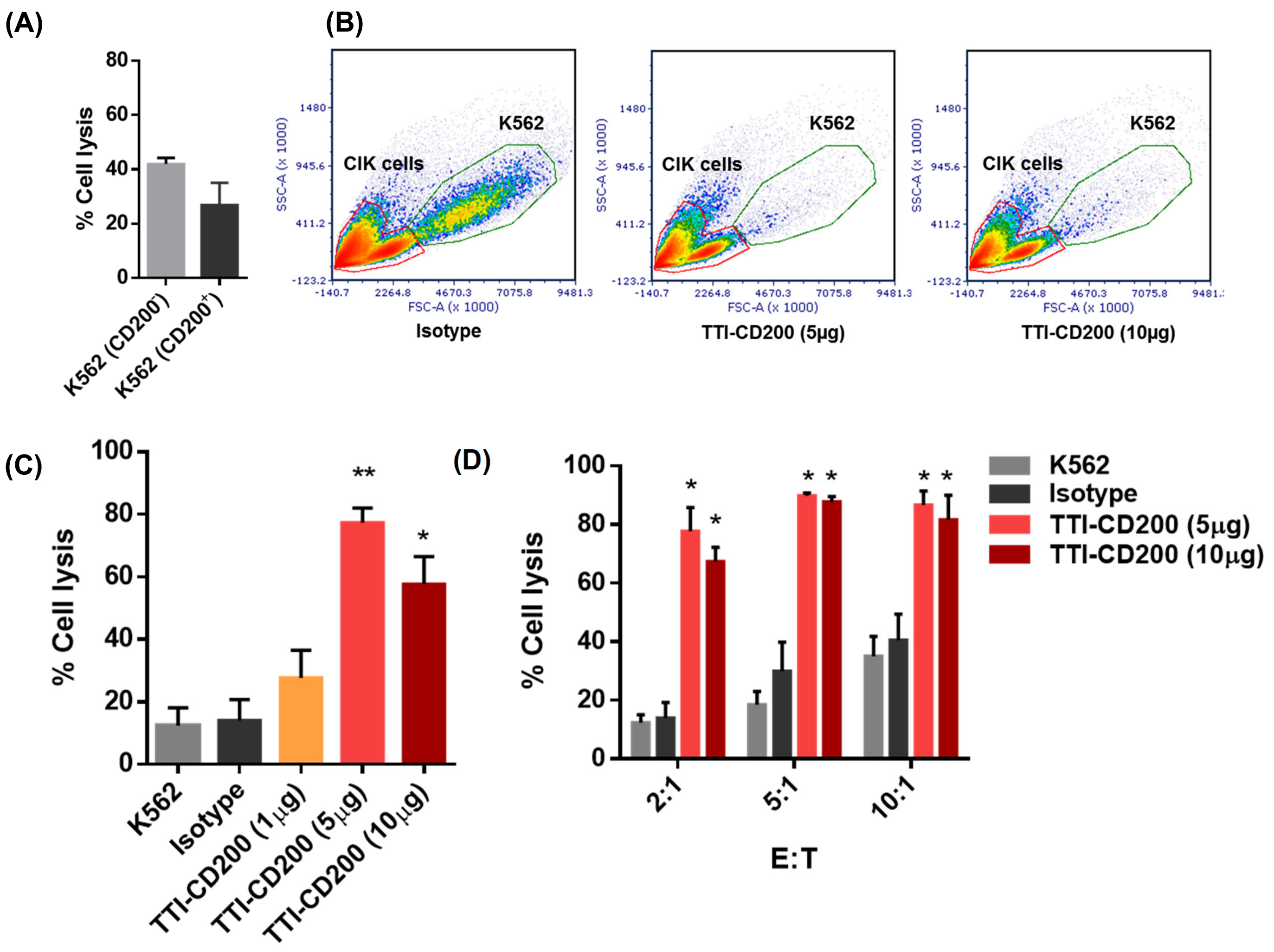

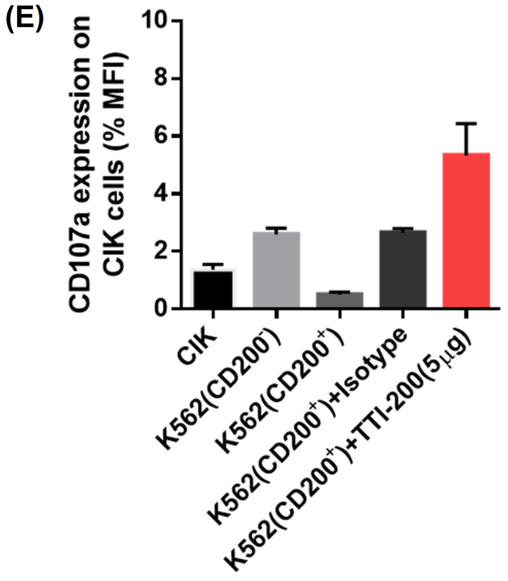

Supplementary Fig. S4. TTI-CD200 treatment enhances CIK mediated lysis in AML cells in vitro. (A) Bar graphs indicating percent cell lysis of K562 (CD200$^-$) parental cells in comparison with K562 overexpressing CD200 by CIK cells at Effector to Target ratio (E:T) of 10:1. (B) Flow cytometric plots showing the cell lysis of K562(CD200$^+$) towards CIK cells in the presence of either TTI-CD200 (5 and 10μg) or Isotype (10μg) with E:T of 10:1. Red and green gates respectively defines CIK and K562 cells. (C) Bar graphs representing percent cell lysis of K562 (CD200$^+$) cells in the presence of different concentrations of TTI-CD200 (1, 5, 10μg) or Isotype. (D) Bar graphs representing percent cell lysis of K562 (CD200$^+$) cells towards CIK cells at different E:T ratio of 2:1, 5:1 and 10:1. Cells were pretreated for 1h with TTI-CD200 (5μg and 10μg) or Isotype (10μg). (E) Bar graphs depicting CD107a expression (as percent MFI) on the surface of CIK cells co-cultured with K562 (CD200$^+$) cells. CD107a expression represented increased NK-like activity of CIK cells in the presence of TTI-CD200 (5μg) as compared to Isotype control. All data sets representative of three independent experiments where bar graphs represent mean ±1SD. Statistical significance denoted by *$p \leq 0.05$ or ** $p \leq 0.01$ analysed by Tukey's multiple comparison test.

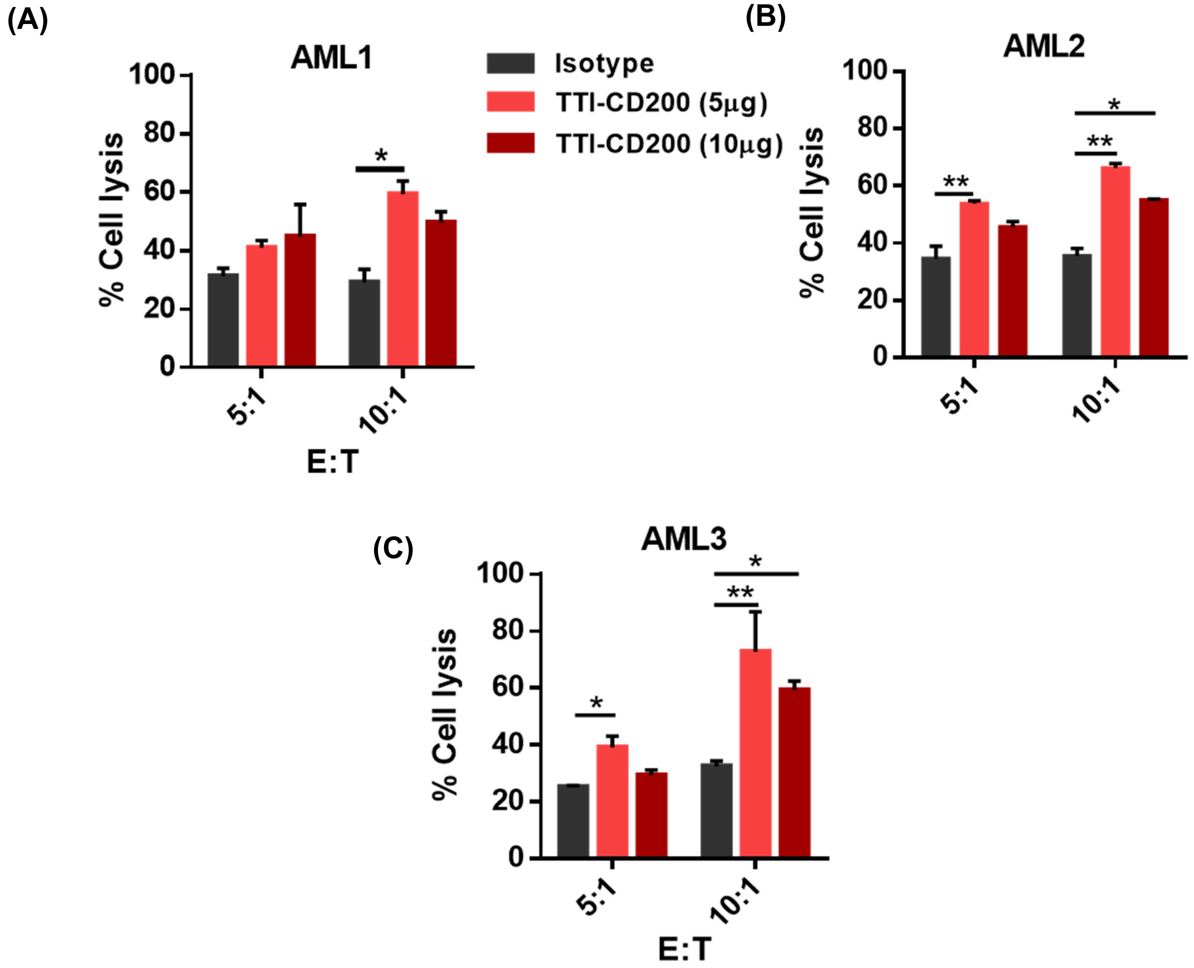

Supplementary Fig. S5. CD200 blocking increases CIK mediated cell lysis in AML blasts. Bar graphs indicating percent cell lysis of primary AML blasts towards CIK cells. Blasts were pre-treated with either TTI-CD200 (5 and 10μg) or isotype (10μg) for 1h followed by incubation with CIK cells for 5h at two E:T ratios of 5:1 and 10:1. Data are represented as mean ± 1SD from two independent experiments. Statistical significance denoted by *p≤ 0.05 or ** p≤ 0.01 analysed by Dunnett's multiple comparison test.

References

1. Tonks A, Pearn L, Musson M, Gilkes A, Mills KI, Burnett AK, et al. Transcriptional dysregulation mediated by RUNX1-RUNX1T1 in normal human progenitor cells and in acute myeloid leukaemia. *Leukemia*. 2007;**21**:2495-505.

2. Coles SJ, Gilmour MN, Reid R, Knapper S, Burnett AK, Man S, et al. The immunosuppressive ligands PD-L1 and CD200 are linked in AML T-cell immunosuppression: identification of a new immunotherapeutic synapse. *Leukemia*. 2015;**29**:1952-4.

3. Coles SJ, Wang EC, Man S, Hills RK, Burnett AK, Tonks A, et al. CD200 expression suppresses natural killer cell function and directly inhibits patient anti-tumor response in acute myeloid leukemia. *Leukemia*. 2011;**25**:792-9.

4. Coles SJ, Hills RK, Wang EC, Burnett AK, Man S, Darley RL, et al. Expression of CD200 on AML blasts directly suppresses memory T-cell function. *Leukemia*. 2012;**26**:2148-51.

5. Pizzitola I, Anjos-Afonso F, Rouault-Pierre K, Lassailly F, Tettamanti S, Spinelli O, et al. Chimeric antigen receptors against CD33/CD123 antigens efficiently target primary acute myeloid leukemia cells in vivo. *Leukemia*. 2014;**28**:1596-605.
